# Value of the fT3/fT4 ratio and its combination with the GRACE risk score in predicting the prognosis in euthyroid patients with acute myocardial infarction undergoing percutaneous coronary intervention: a prospective cohort study

**DOI:** 10.1186/s12872-018-0916-z

**Published:** 2018-09-10

**Authors:** Tongtong Yu, Chunyang Tian, Jia Song, Dongxu He, Jiake Wu, Zongyu Wen, Zhijun Sun, Zhaoqing Sun

**Affiliations:** Department of Cardiology, Shengjing Hospital of China Medical University, Shenyang, Liaoning People’s Republic of China

**Keywords:** fT3/fT4 ratio, GRACE risk score, Acute myocardial infarction, Percutaneous coronary intervention

## Abstract

**Background:**

Thyroid hormones deeply influence the cardiovascular system; however, the association between the fT3/fT4 ratio and the clinical outcome in euthyroid patients with acute myocardial infarction (AMI) undergoing percutaneous coronary intervention (PCI) is not well defined. Therefore, the present study aimed to assess the prognostic performance of the fT3/fT4 ratio in predicting the long-term prognosis in euthyroid patients with AMI undergoing PCI.

**Methods:**

In a prospective cohort study with a 1-year follow-up, according to the clinical end point, 953 euthyroid individuals (61.0 ± 11.6; female, 25.8%) were divided into two groups: (1) the survival group (*n* = 915) and (2) the death group (*n* = 38).

**Results:**

According to Cox regression multivariate analysis, fT4 (HR: 1.249, 95% CI: 1.053–1.480, *p* = 0.010) and the fT3/fT4 ratio (HR: 3.546, 95% CI: 1.705–7.377, *p* = 0.001) were associated with an increased risk of 1-year all-cause mortality. The prognostic performance of the fT3/fT4 ratio was similar to the Global Registry of Acute Coronary Events (GRACE) score in predicting 1-year all-cause mortality (C-statistic: *z* = 0.261, *p* = 0.794; IDI: -0.017, *p* = 0.452; NRI: -0.049, *p* = 0.766), but better than fT4 (C-statistic: *z* = 2.438, *p* = 0.015; IDI: 0.053, *p* = 0.002; NRI: 0.656, *p* < 0.001). The fT3/fT4 ratio also significantly improved the prognostic performance of the GRACE score (GRACE score vs GRACE score + fT3/fT4 ratio: C-statistic: *z* = 2.116, *p* = 0.034; IDI: 0.0415, *p* = 0.007; NRI: 0.614, *p* < 0.001).

**Conclusions:**

In euthyroid patients with AMI undergoing PCI, the fT3/fT4 ratio was an independent predictor of 1-year all-cause mortality. Its prognostic performance was similar to the GRACE score, and also improved its prognostic performance (GRACE score vs GRACE score + fT3/fT4 ratio).

## Background

Despite the use of novel treatment strategies, patients with acute myocardial infarction (AMI) still suffer from an adverse prognosis [1]. Attention should still focus on revealing novel treatment targets and important risk factors. In AMI, serum triiodothyronine (T3) is decreased [2–5], while serum thyroxine (T4) remains almost unchanged [2–4] or declines [5]. In fact, the cardiovascular system is the foremost target of thyroid hormones, and is adversely affected even if these hormone levels only change slightly [6]. A decrease in serum T3 has been found to be a predictor of larger myocardial injury size [4, 5, 7], worse cardiac function [8, 9], greater thrombus burden [10], and a poorer prognosis [5, 11–14] in AMI. A recent study also confirmed the association between free T4 and adverse outcomes in acute coronary syndrome [15]. Serum T3, which is the most important bioactive thyroid hormone for cardiomyocytes, is mostly produced by the peripheral process of deiodination of T4 [6]. In AMI, the studies have suggested that the peripheral conversion of T4 into T3 was reduced [3, 4]. However, no previous study has focused on the clinical value of the disturbance of the conversion of T4 into T3 in patients with AMI. The fT3/fT4 ratio, a thyroid hormone index, could reflect deiodinase activity [16], and thus, represent the conversion of T4 to T3 [17]. The Global Registry of Acute Coronary Events (GRACE) score is widely recommended to calculate in-hospital and long-term mortality in acute coronary syndrome (ACS), which helps clinical decision-making and discriminates high-risk patients [18–22]. The GRACE score has passed rigorous validation since its conception in 2004; however, several changes has been approved in the diagnostic and management tools of ACS in the last 14 years. Moreover, the estimation of risk is a continuous process, and further refinement of current risk scores may help the decision-making process in real world practice. Furthermore, novel risk factors are not included in the GRACE score, such as thyroid hormone-related indicators including thyrotropin, fT3, fT4 and the fT3/fT4 ratio.

In the present study, we aimed to assess whether the fT3/fT4 ratio is a useful clinical parameter in predicting long-term prognosis in euthyroid patients with AMI undergoing PCI. In addition, we compared the prognostic performance of fT3, fT4, and the fT3/fT4 ratio using the GRACE score as the reference standard. Moreover, we confirmed whether fT3, fT4, and the fT3/fT4 ratio could improve the prognostic performance of the GRACE score.

## Methods

### Study design and setting

The present study was based on a prospective cohort. From January 1st 2015 to July 31st 2016, 1195 consecutive patients with AMI were hospitalized and underwent successful PCI at a large-scale hospital in Northeast China (Shengjing Hospital of China Medical University, Shenyang, China). AMI included non-ST-segment elevation myocardial infarction (NSTEMI) and ST-segment elevation myocardial infarction (STEMI). NSTEMI is defined as chest discomfort or anginal equivalent, ST-segment depression, transitory ST-segment elevation or prominent T-wave inversion, and positive cardiac biomarkers (CKMB, T/I troponin) [18–21]. STEMI is defined as chest pain and significant ST-segment elevation (≥ 0.1 mV in at least 2 standard leads or ≥ 0.2 mV in at least 2 contiguous precordial leads) or new left bundle branch block [18–21]. PCI was performed in accordance with current guidelines [18–21]. The duration of dual antiplatelet therapy was at least 12 months [18–21, 23]. Clinical and procedural data from all cases were collected by the investigators from electronic medical records. On admission, venous blood samples were drawn in standard tubes at room temperature, rapidly centrifuged, and the levels of thyrotropin (TSH), free T3 (fT3), and free T4 (fT4) were measured using a completely automated immunoassay analyzer (i2000, Abbott, USA) in the core laboratory of Shengjing Hospital. The reference intervals of our laboratory were as follows: TSH, 0.3–4.8 uIu/mL; fT3, 2.63–5.71 pmol/L; and fT4, 9.01–19.05 pmol/L. Patients with circulating levels of TSH, fT3, and fT4 all in the reference range were defined as euthyroid. Prospective clinical follow-up after discharge was performed regularly in all cases by direct hospital visits and telephone interviews with the patient’s general practitioner/cardiologist, the patient, or the patient’s family. All events were adjudicated and classified by two cardiologists. Exclusion criteria included: (1) no thyroid or GRACE score data (13 cases); (2) primary hypothyroidism or hyperthyroidism (27 cases); (3) subhypothyroidism or subhyperthyroidism or low T3 syndrome (132 cases); (4) any other abnormal thyroid status (43 cases); (5) concomitant treatment with synthetic thyroid hormones, antithyroid drugs, corticosteroids, dopamine, dobutamine, or amiodarone (7 cases); or (6) loss of follow-up (20 cases). Finally, the present study included 953 euthyroid patients with AMI undergoing PCI, all of whom underwent a 1-year follow-up. The clinical endpoint of the study was 1-year all-cause mortality. All patients were divided into two groups: (1) the survival group (*n* = 915, 96.0%) and (2) the death group (*n* = 38, 4.0%). The present study complies with the Declaration of Helsinki; and Shengjing Hospital of China Medical University Research Ethics Committee approved the research protocol. Written informed consent was formally obtained from all participants.

### Statistical analysis

The cumulative event rate was estimated from Kaplan-Meier curves and compared using the log-rank test. The Cox proportional-hazards regression model was used to analyze the effects of the variables on event-free survival. The variables that showed significance in univariate analysis (Table 1, *p* < 0.1) entered the final model. Results are reported as hazard ratios (HRs) with associated 95% confidence intervals (CIs). The predictive performance of fT3, fT4, the fT3/fT4 ratio, and the GRACE score was assessed by indices of discrimination (C-statistic). As continuous variables, the predictive performance of the GRACE score, the GRACE score + fT3, the GRACE score + fT4, and the GRACE score + the fT3/fT4 ratio was assessed by indices of discrimination (C-statistic), calibration (the Hosmer−Lemeshow (HL) test and Nagelkerke−R^2^), and precision (Brier scores). The C-statistic was compared using a nonparametric test developed by DeLong et al. [24]. Each model was entered into a logistic regression model to obtain the individual risk probability of all-cause death. The HL test and the Nagelkerke−R^2^ from the regression model was used as an indicator of the goodness-of-fit of each risk model and to assess their calibration ability [25]. As continuous variables, Brier scores of the fT3 + GRACE, fT4 + GRACE, fT3/fT4 ratio + GRACE, and GRACE scores were also calculated [26]. Moreover, we used absolute integrated discrimination improvement (IDI) and category-free net reclassification improvement (NRI) to evaluate improvements in risk prediction quantitation of the fT3 + GRACE, fT4 + GRACE, fT3/fT4 ratio + GRACE, and GRACE scores as continuous variables [27]. All tests were two-sided, and the statistical significance is defined as *p* < 0.05. All statistical analyses were performed using the Statistical Analysis System version 9.4 (SAS, SAS Institute Inc., Cary, North Carolina, USA).

**Table 1:** Baseline Characteristics of the study population, median (IQR), or N (%), or means±SD

Variable	All Patients (*n* = 953)	Survival Group (*n* = 915)	Death Group (*n* = 38)	*p* value
Demographics				
Age, yrs	61.0 ± 11.6	60.8 ± 11.6	67.6 ± 10.6	<0.001
Female	246 (25.8)	231 (25.2)	15 (39.5)	0.050
Medical history				
History of Diabetes Mellitus	281 (29.5)	269 (29.4)	12 (31.6)	0.773
History of Hypertension	533 (55.9)	502 (54.9)	31 (81.6)	0.001
History of MI	107 (11.2)	102 (11.1)	5 (13.2)	0.701
Prior PCI	90 (9.4)	89 (9.7)	1 (2.6)	0.143
Presentation				
Killip class III/IV on admission	19 (2.0)	11 (1.2)	8 (30.0)	<0.001
SBP on admission, mm Hg	134.4 ± 22.7	134.3 ± 22.6	138.5 ± 26.2	0.259
Heart rate on admission, bpm	76.6 ± 14.7	76.2 ± 14.3	86.4 ± 20.4	<0.001
GRACE score	127.7 ± 32.2	126.5 ± 31.6	158.0 ± 32.7	<0.001
Diagnosis on admission				0.573
STEMI	519 (54.5)	500 (54.6)	19 (50.0)	
NSTEMI	434 (45.5)	415 (45.4)	19 (50.0)	
Troponin-I on admission, ng/mL	4.06 (0.38, 31.77)	4.04 (0.37, 32.07)	5.31 (0.67, 25.01)	0.870
Creatinine on admission, umol/l	72 (61, 86)	72 (61, 86)	81 (62, 107)	0.090
Albumin on admission, g/L	39.4 ± 3.6	39.5 ± 3.5	37.5 ± 4.2	0.001
TSH, uIU/mL	1.546 ± 0.961	1.549 ± 0.965	1.485 ± 0.853	0.687
fT3, pmol/L	3.902 ± 0.588	3.913 ± 0.581	3.659 ± 0.694	0.009
fT4, pmol/L	13.11 ± 1.84	13.08 ± 1.82	14.02 ± 2.20	0.002
fT3/ fT4 ratio	0.302 ± 0.055	0.304 ± 0.054	0.262 ± 0.058	<0.001
Percutaneous coronary intervention details				
Left main disease	75 (7.9)	71 (7.8)	4 (10.5)	0.535
Three-vessel disease	242 (25.4)	232 (25.4)	10 (26.3)	0.894
TIMI flow grade 0/1 on arrival	741 (77.8)	714 (78.0)	27 (71.1)	0.311
TIMI flow grade 3 post PCI	947 (99.4)	910 (99.5)	37 (97.4)	0.111
Medical treatment at discharge				
Aspirin	950 (99.7)	912 (99.7)	38 (100)	0.724
Clopidogrel	865 (90.8)	830 (90.7)	35 (92.1)	0.771
Ticagrelor	75 (7.9)	72 (7.9)	3 (7.9)	0.995
Statin	943 (99.0)	905 (98.9)	38 (100)	0.517
ACEI/ARB	510 (53.5)	490 (53.6)	20 (52.6)	0.911
Beta-blockers	481 (50.5)	461 (50.4)	20 (52.6)	0.786

*MI* myocardial infarction, *PCI* percutaneous coronary intervention, *SBP* systolic blood pressure, *bpm* beats per minute, *STEMI* ST-segment elevation myocardial infarction, *NSTEMI* non-ST-segment elevation myocardial infarction, *TSH* thyrotropin, *fT3* free triiodothyronine, *fT4* free thyroxine, *ACEI/ARB* Angiotensin-converting enzyme inhibitors / Angiotensin receptor blockers

## Results

### Baseline characteristics

**Fig. 1:**
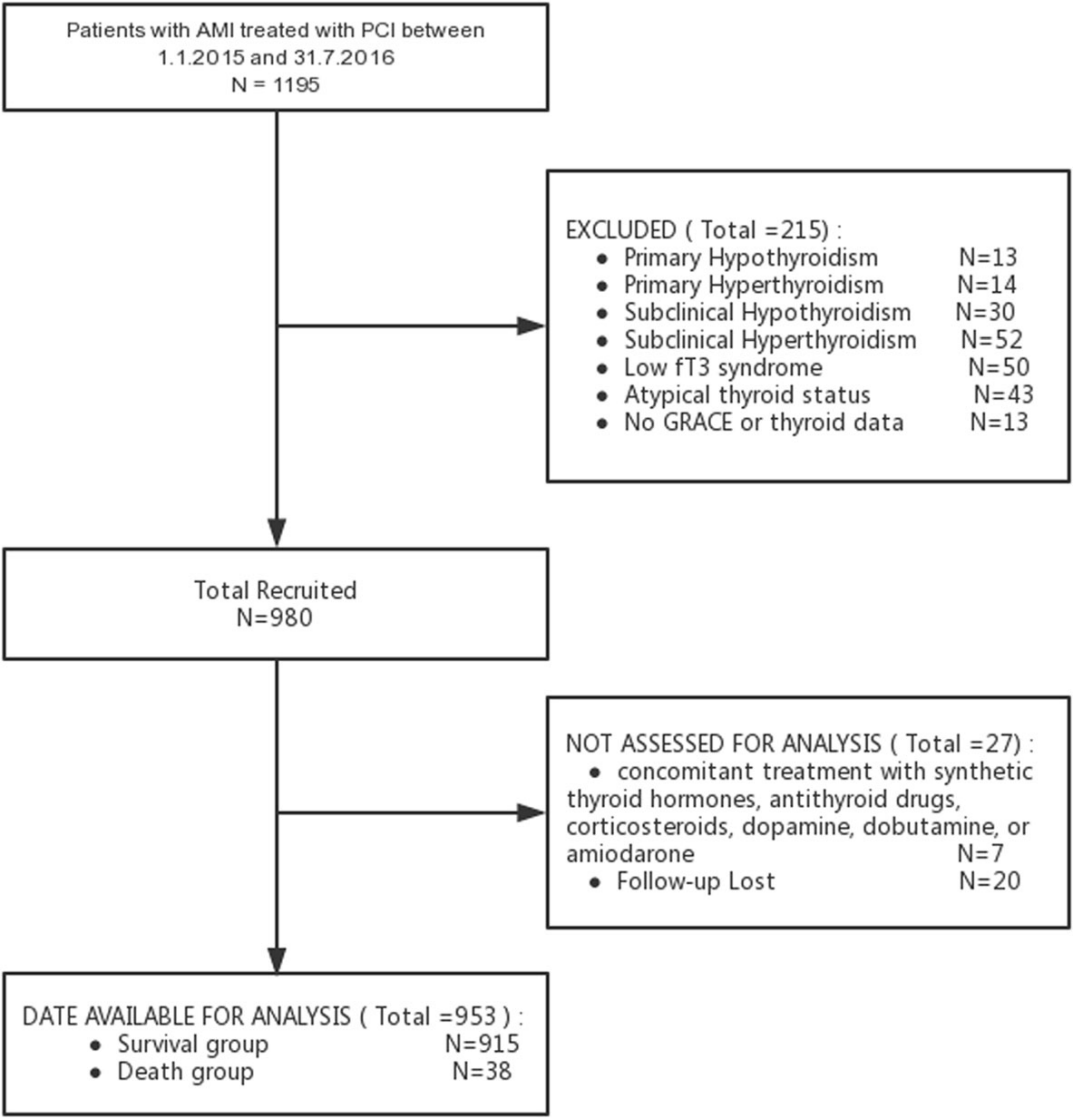
Flow diagram of participant selection

Figure 1 represents the flowchart of patient selection. The final study cohort consisted of 953 euthyroid patients with AMI undergoing PCI. The cohort was divided into two groups: (1) the survival group (915 patients (96.0%)) and (2) the death group (38 patients (4.0%)). The clinical characteristics of the two groups are shown in Table 1. Patients in the death group were older and had a significantly higher heart rate on admission, GRACE score, albumin on admission, and fT4; and a lower fT3 and fT3/fT4 ratio, as compared with those in the survival group (Table 1). The rates of hypertension and Killip class III/IV on admission were significantly higher in the death group (Table 1).

### Prognostic performance of different thyroid hormone-related indicators in prognosis prediction

The univariate analysis revealed that fT3, fT4, and fT3/fT4 ratio, but not TSH, were associated with 1-year all-cause mortality (Table 2). In Cox regression multivariate analysis, fT4 and the fT3/fT4 ratio remained associated with 1-year all-cause mortality; 24.9% per pmol/L increase in fT4 concentration (HR: 1.249, 95% CI: 1.053–1.480, *p* = 0.010) and a 2.546-fold per 0.1 unit decrease in the fT3/fT4 ratio (HR: 3.546, 95% CI: 1.705–7.377, *p* = 0.001) (Table 2).

**Table 2:** Effects of multiple variables on Clinical Outcomes in Univariate and Multivariate Analysis

	Univariate Analysis			Multivariate Analysis		
	HR	95% CI	*p* value	HR	95% CI	*p* value
TSH (per 1 uIU/mL increase)	0.930	0.660–1.311	0.680			
fT3 (per 1 pmol/L decrease)	2.154	1.207–3.842	0.009	0.887	0.463–1.700	0.718 ^a^
fT4 (per 1 pmol/L increase)	1.282	1.095–1.499	0.002	1.249	1.053–1.480	0.010 ^a^
fT3/fT4 ratio (per 0.1 unit decrease)	6.742	3.534–12.859	<0.001	3.546	1.705–7.377	0.001 ^a^

^a^Adjusted for age, gender, history of hypertension, Killip class III/IV on admission, Heart rate on admission, creatinine on admission, albumin on admission

**Fig. 2:**
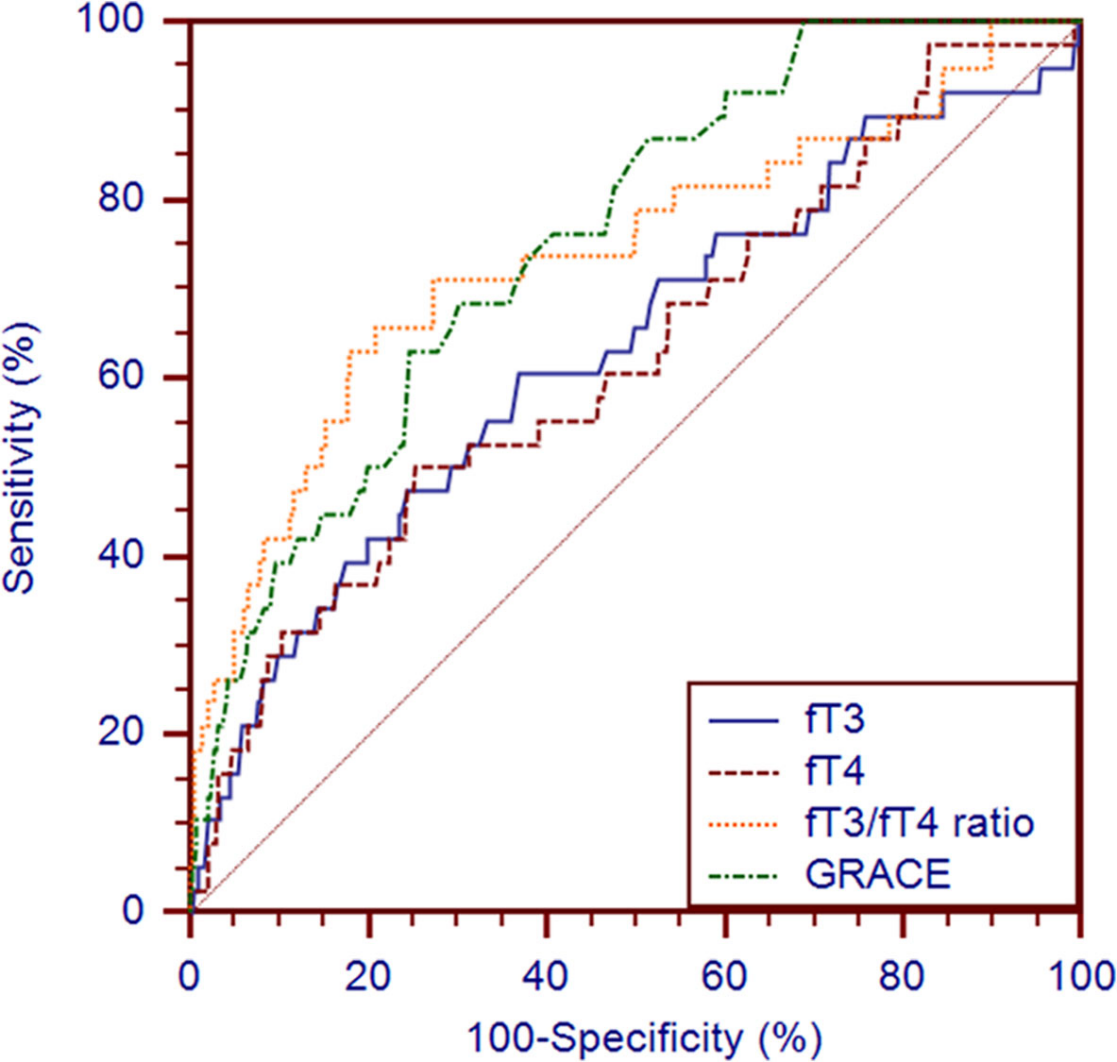
Receiver operating characteristic curves of fT3, fT4, fT3/fT4 ratio and GRACE for 1-year all-cause death prediction

The C-statistic of fT3, fT4, the fT3/fT4 ratio, and the GRACE score in predicting all-cause mortality was 0.631 (95% CI: 0.600–0.662), 0.624 (95% CI: 0.592–0.655), 0.738 (95% CI: 0.709–0.766), and 0.755 (95% CI: 0.727–0.782), respectively (Table 3 and Fig. 2). The cut-off values for fT3, fT4, and the fT3/fT4 ratio were: 3.685 with a sensitivity of 0.605 and a specificity of 0.632; 14.21 with a sensitivity of 0.500 and a specificity of 0.748; and 0.255 with a sensitivity of 0.632 and a specificity of 0.820, respectively.

**Table 3:** C-statistic of different parameters for clinical outcomes prediction

	C-statistic	95% CI	*p* value
fT3	0.631	0.600–0.662	0.006
fT4	0.624	0.592–0.655	0.010
fT3/fT4 ratio	0.738	0.709–0.766	<0.001
GRACE	0.755	0.727–0.782	<0.001

**Fig. 3:**
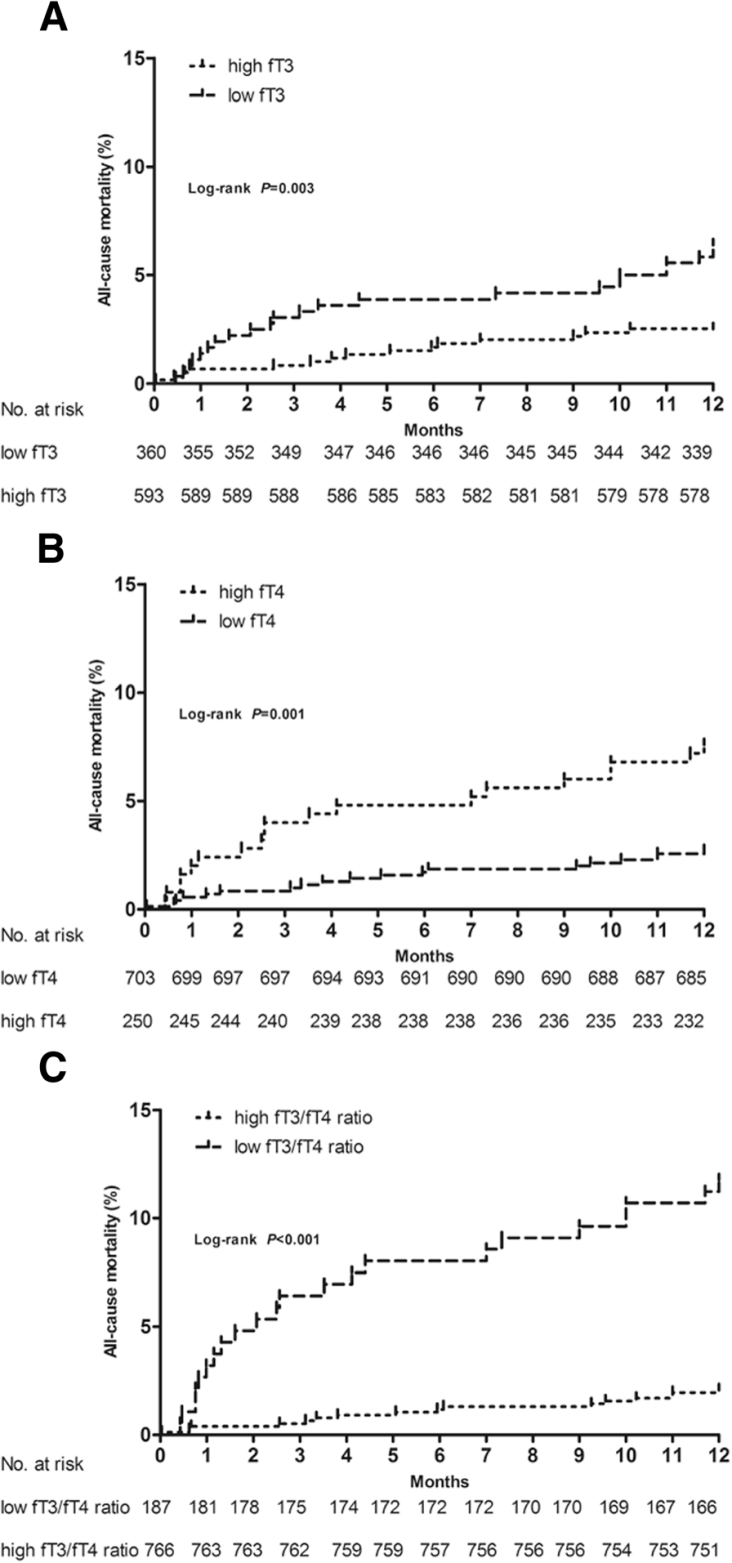
Kaplan-Meier survival curves for 1-year all-cause death by the cut off values for (**a**) fT3, **b** fT4 and (**c**) fT3/fT4 ratio (high fT3 group: fT3>3.685 pmol/L and low fT3 group: fT3 ≤ 3.685 pmol/L; high fT4 group: fT4 ≥ 14.21 pmol/L and low fT4 group: fT4<14.21 pmol/L; high fT3/fT4 ratio group: fT3/fT4 ratio>0.255 and low fT3/fT4 ratio group: fT3/fT4 ratio ≤ 0.255)

Based on the cut-off value for fT3, the cohort was divided into two groups: the high fT3 group (fT3 > 3.685 pmol/L, *n* = 593) and the low fT3 group (fT3 ≤ 3.685 pmol/L, *n* = 360. The unadjusted Kaplan-Meier estimate for all-cause mortality was significantly higher in the low fT3 group as compared with the high fT3 group (all-cause mortality: 6.4% vs 2.5%, *p* = 0.003) (Fig. 3a). Based on the cut-off value for fT4, the cohort was divided into two groups: the high fT4 group (fT4 ≥ 14.21 pmol/L, *n* = 250) and the low fT4 group (fT4 < 14.21 pmol/L, *n* = 703). The unadjusted Kaplan-Meier estimate for all-cause mortality was significantly higher in the high fT4 group as compared with the low fT4 group (all-cause mortality: 7.6% vs 2.7%, *p* = 0.001) (Fig. 3b). Based on the cut-off value for the fT3/fT4 ratio, the cohort was divided into two groups: the high fT3/fT4 ratio group (fT3/fT4 ratio > 0.255, *n* = 766) and the low fT3/fT4 ratio group (fT3/fT4 ratio ≤ 0.255, *n* = 187). The unadjusted Kaplan-Meier estimate for all-cause mortality was significantly higher in the low fT3/fT4 ratio group as compared with the high fT3/fT4 ratio group (all-cause mortality: 11.8% vs 2.1%, *p* < 0.001) (Fig. 3c).

### Comparison of the prognostic performance of fT3, fT4, the fT3/fT4 ratio, and the GRACE score in prognosis prediction

The prognostic performance of the fT3/fT4 ratio was similar to that of the GRACE score in predicting 1-year all-cause mortality (C-statistic: *z* = 0.261, *p* = 0.794; IDI: -0.017, *p* = 0.452; NRI: -0.049, *p* = 0.766), but better than that of fT3 (C-statistic: *z* = 2.062, *p* = 0.039; IDI: 0.056, *p* = 0.001; NRI: 0.625, *p* < 0.001) and fT4 (C-statistic: *z* = 2.438, *p* = 0.015; IDI: 0.053, *p* = 0.002; NRI: 0.656, *p* < 0.001) (Table 4).

**Table 4:** Comparisons of the predictive performance of fT3, fT4, fT3/fT4 ratio and GRACE for the prognosis prediction

	*z* for C-statistic	*p* for C-statistic	NRI	*p* for NRI	IDI	*p* for IDI
fT3 vs. fT3/fT4 ratio	2.062	0.039	0.625	<0.001	0.056	0.001
fT4 vs. fT3/fT4 ratio	2.438	0.015	0.656	<0.001	0.053	0.002
GRACE vs. fT3/fT4 ratio	0.261	0.794	-0.049	0.766	-0.017	0.452
fT3 vs. GRACE	2.013	0.044	0.594	<0.001	0.039	0.005
fT4 vs. GRACE	2.202	0.028	0.531	0.001	0.036	0.011
fT3 vs. fT4	0.117	0.907	−0.075	0.649	−0.002	0.677

### Improvement in the prognostic performance of the GRACE score when combined with thyroid hormone-related indicators

**Fig. 4:**
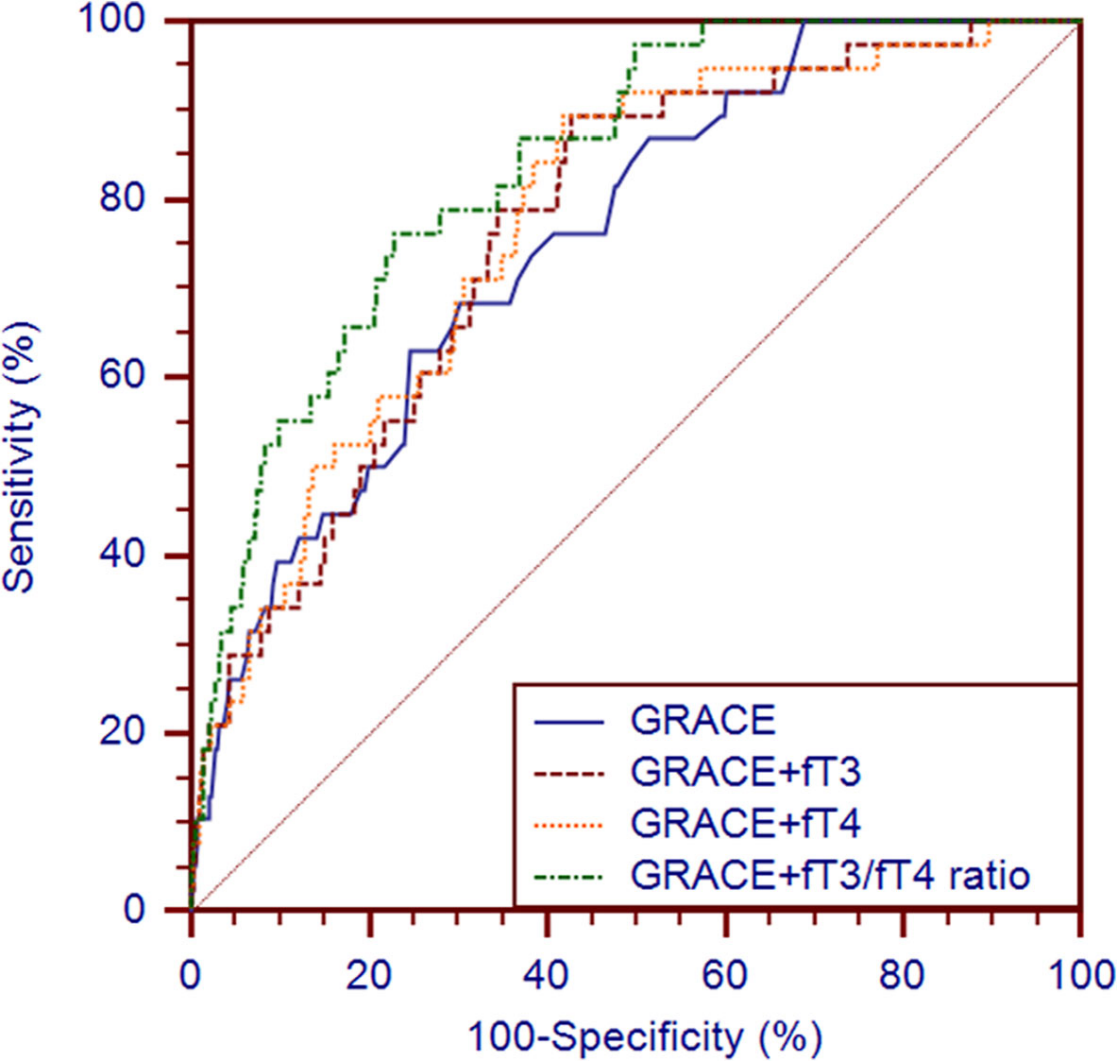
Receiver operating characteristic curves of GRACE+fT3, GRACE+fT4, GRACE+fT3/fT4 ratio and GRACE for 1-year all-cause death prediction

The C-statistic of the GRACE score, the GRACE score + fT3, the GRACE score + fT4, and the GRACE score + the fT3/fT4 ratio in predicting all-cause mortality was 0.755 (95% CI: 0.727–0.782), 0.765 (95% CI: 0.736–0.791), 0.775 (95% CI: 0.747–0.801), and 0.836 (95% CI: 0.811–0.859), respectively (Table 5 and Fig. 4). Among the four models, the HL *p*-value of the GRACE score was the highest; the Nagelkerke-R^2^ of the GRACE score + the fT3/fT4 ratio was the highest; and the Brier score of the GRACE score + the fT3/fT4 ratio was the lowest (Table 5). However, only the new model in which the GRACE score was combined with the fT3/fT4 ratio improved the prognostic performance, which was better than that of the GRACE score (C-statistic: *z* = 2.116, *p* = 0.034; IDI: 0.0415, *p* = 0.007; NRI: 0.614, *p* < 0.001). In contrast, the prognostic performance of the GRACE score + fT3 and the GRACE score + fT4 was similar to that of the GRACE score (C-statistic: *z* = 0.608, *p* = 0.543; IDI: 0.0047, *p* = 0.277; NRI: 0.198, *p* = 0.231; C-statistic: *z* = 1.078, *p* = 0.281; IDI: 0.0095, *p* = 0.142; NRI: 0.243, *p* = 0.141, respectively) (Table 6).

**Table 5:** GRACE, GRACE+ fT3/fT4 ratio, GRACE+ fT3 and GRACE+ fT4 performance for the prognosis prediction

	Discrimination				Calibration		Precision
	C-statistic	Standard error	*p* value	95% CI	HL *p*-Value	R^2^	Brier Score
GRACE	0.755	0.0364	<0.001	0.727–0.782	0.337	0.119	0.0366
GRACE+ fT3/fT4 ratio	0.836	0.0286	<0.001	0.811–0.859	0.180	0.157	0.0348
GRACE+ fT3	0.765	0.0359	<0.001	0.736–0.791	0.185	0.090	0.0364
GRACE+ fT4	0.775	0.0358	<0.001	0.747–0.801	0.029	0.101	0.0362

**Table 6:** Comparisons of the predictive performance of GRACE, GRACE+ fT3/fT4 ratio, GRACE+ fT3 and GRACE+ fT4 for the prognosis prediction

	*z* for C-statistic	*p* for C-statistic	NRI	*p* for NRI	IDI	*p* for IDI
GRACE vs. GRACE+ fT3/fT4 ratio	2.116	0.034	0.614	<0.001	0.0415	0.007
GRACE vs. GRACE+ fT3	0.608	0.543	0.198	0.231	0.0047	0.277
GRACE vs. GRACE+ fT4	1.078	0.281	0.243	0.141	0.0095	0.142

## Discussion

The present study tested the association between the fT3/fT4 ratio and the long-term prognosis in euthyroid patients with AMI undergoing PCI. The main findings were as follows: (1) the fT3/fT4 ratio was an independent predictor of 1-year all-cause mortality; (2) the prognostic performance of the fT3/fT4 ratio was similar to that of the GRACE score, and better than that of fT3 and fT4; and (3) only the fT3/fT4 ratio could improve the prognostic performance of the original GRACE score model.

Thyroid hormones extensively affect the physiological and pathological processes of the cardiovascular system [6]. Previous studies have demonstrated that even mild thyroid dysfunction in cardiac patients results in an adverse prognosis: subclinical hypothyroidism is a strong indicator of atherosclerosis risk [28, 29]; subclinical hyperthyroidism is associated with an increased risk of atrial fibrillation [30]; a mildly altered thyroid status (including subclinical hypothyroidism, subclinical hyperthyroidism, and low T3 syndrome) is also associated with an increased risk of mortality in patients with cardiac disease [14, 31–33]. T3 and T4 are two main iodinated hormones secreted by the thyroid gland. Since the affinity of the thyroid hormone receptors is far higher for T3 than for T4, T3 is considered the biologically active hormone, and T4 must be converted to T3 to produce potent thyroid hormone receptor­mediated effects [6]. Less than 20% of circulating T3 is directly secreted by the thyroid gland, while more than 80% is produced by a peripheral process of deiodination of T4 [6]. Thus, the conversion of T4 to T3 is very important in the production of circulating T3 and the thyroid hormone action on the heart. In chronic and acute illness, this conversion has been reported to decline [3, 4, 34]. Furthermore, a disturbance in the conversion of T4 to T3 contributes to reduced T3 production in low T3 syndrome and its etiology [16, 34, 35]. Previous studies have demonstrated that the fT3/fT4 ratio could reflect deiodinase activity [16], and thus, represent the conversion of T4 to T3 [17]. A significant correlation between the fT3/fT4 ratio and infarct size has been observed [4]; however, no study has focused on the clinical value of a disturbance in the conversion of T4 to T3 in AMI patients. The present study demonstrates that in euthyroid patients with AMI undergoing PCI, the fT3/fT4 ratio was associated with 1-year all-cause mortality (all-cause mortality for low the fT3/fT4 ratio group vs the high fT3/fT4 ratio group: 11.8% vs 2.1%, *log-rank* test: *p* < 0.001). The results of Cox regression multivariate analysis further confirmed that a reduction in the fT3/fT4 ratio was associated with a 2.546-fold greater likelihood of 1-year all-cause death. The discriminative performance of the fT3/fT4 ratio was encouraging (C-statistic: 0.738; 95% CI: 0.709–0.766), far better than that of fT3 and fT4, and similar to that of the GRACE score in predicting 1-year all-cause mortality in euthyroid patients with AMI undergoing PCI. Taken together, the fT3/fT4 ratio is a very useful clinical parameter in predicting long-term prognosis in euthyroid patients with AMI undergoing PCI, can help risk stratification in AMI patients, and identify those patients at high risk of 1-year all-cause death. Therefore, the fT3/fT4 ratio may be taken as a better risk factor for AMI; however, further large cohort studies are needed in this regard.

The GRACE score, containing the main traditional risk factors for cardiovascular disease, was derived in the early twenty-first century. Since then, increasing amounts of novel risk factors have been studied; nevertheless, the GRACE score does not contain any of these new risk factors such as thyroid hormone-related indicators, including thyrotropin, fT3, fT4, and the fT3/fT4 ratio [6]. The present study found that the fT3/fT4 ratio was a valid adjunct to the GRACE score. The new model, the GRACE score + the fT3/fT4 ratio, showed good discrimination (C-statistic: 0.836), calibration (HL *p*-value: 0.180, R^2^: 0.157), and precision (Brier score: 0.0348). The prognostic performance of the new model was also better than that of the original model (only the GRACE score). In clinical practice, the new model, the GRACE score combined with the fT3/fT4 ratio, can also help make a more accurate assessment of the long-term mortality risk and more precise clinical decisions.

The present study has several limitations. Firstly, this was a single center, observational study; thus, potential confounders and selection bias could not be completely adjusted, since some important clinical data were collected from electronic medical records. However, it has the advantage of being a prospective study. Secondly, thyroid function tests were not repeated within 2–12 weeks to exclude transient forms of thyroid dysfunction as recommended by the guidelines [36], at euthyroid diagnosis. Thirdly, previous studies have indicated that iodinated contrast media may influence thyroid function [37, 38]; however, in the present study, the thyroid function of some patients was tested following the use of iodinated contrast media, since they needed emergency PCI. Fourthly, the present study did not test total T3 (TT3) and total T4 (TT4) levels, since only free T3 and free T4 can enter target cells and play a role, directly reflecting the state of thyroid function [6]. Fifthly, previous studies have found that reverse T3 increased in AMI [2–5], and that increased levels of reverse T3 were also independently associated with 1-year mortality [39]; however, the present study did not test reverse T3 (rT3). In the future, other studies should be performed to obtain the association between prognosis and more thyroid hormone-related indicators including TSH, TT3, TT4, fT3, fT4, rT3, and the fT3/fT4 ratio. Finally, the present study only included AMI patients in whom successful PCI was performed; thus, the results cannot be generalized to all ACS patients.

## Conclusion

In euthyroid patients with AMI undergoing PCI, the fT3/fT4 ratio was an independent predictor of 1-year all-cause mortality, and could also significantly improve the prognostic performance of the GRACE score.

## Abbreviations

ACEI/ARB: Angiotensin-converting enzyme inhibitors / Angiotensin receptor blockers
ACS: Acute coronary syndrome
AMI: Acute myocardial infarction
bpm: Beats per minute
CI: Confidence interval
fT3: Free serum triiodothyronine
fT4: Thyroxine
GRACE score: Global Registry of Acute Coronary Events score
HL: Hosmer-Lemeshow
HR: Hazard ratio
IDI: Integrated discrimination improvement
NRI: Net reclassification improvement
NSTEMI: Non-ST-segment elevation myocardial infarction
PCI: Percutaneous coronary intervention
SBP: Systolic blood pressure
STEMI: ST-segment elevation myocardial infarction
TSH: Thyrotropin
